# Beyond Homozygosity Mapping: Family-Control analysis based on Hamming distance for prioritizing variants in exome sequencing

**DOI:** 10.1038/srep12028

**Published:** 2015-07-06

**Authors:** Atsuko Imai, Akihiro Nakaya, Somayyeh Fahiminiya, Martine Tétreault, Jacek Majewski, Yasushi Sakata, Seiji Takashima, Mark Lathrop, Jurg Ott

**Affiliations:** 1 Department of Cardiovascular Medicine, Osaka University Graduate School of Medicine, Osaka, Japan; 2 Department of Genome Informatics, Osaka University Graduate School of Medicine, Osaka, Japan; 3 McGill University and Genome Québec Innovation Centre, Montréal, QC H3A 1B1, Canada; 4 Department of Medical Biochemistry, Osaka University Graduate School of Medicine, Osaka, Japan; 5 Institute of Psychology, Chinese Academy of Sciences, Beijing, China 100101; and Rockefeller University, New York, NY 10065, USA

## Abstract

A major challenge in current exome sequencing in autosomal recessive (AR) families is the lack of an effective method to prioritize single-nucleotide variants (SNVs). AR families are generally too small for linkage analysis, and length of homozygous regions is unreliable for identification of causative variants. Various common filtering steps usually result in a list of candidate variants that cannot be narrowed down further or ranked. To prioritize shortlisted SNVs we consider each homozygous candidate variant together with a set of SNVs flanking it. We compare the resulting array of genotypes between an affected family member and a number of control individuals and argue that, in a family, differences between family member and controls should be larger for a pathogenic variant and SNVs flanking it than for a random variant. We assess differences between arrays in two individuals by the Hamming distance and develop a suitable test statistic, which is expected to be large for a causative variant and flanking SNVs. We prioritize candidate variants based on this statistic and applied our approach to six patients with known pathogenic variants and found these to be in the top 2 to 10 percentiles of ranks.

In current sequencing of patients in autosomal recessive (AR) families, candidate disease variants are generally prioritized based on well-known filtering steps^1^,^2^. Homozygosity mapping is also often applied to identify long runs of homozygosity^3^, which may be interpreted as harboring segments of DNA identical by descent (IBD), but length alone is known to be a poor statistic for this purpose^4^. Information from unaffected individuals and estimating haplotype frequencies to identify ancestral haplotypes may aid in the identification of segments of IBD^4^. Here we developed a novel method to prioritize candidate variants in AR families based on direct comparison of segments of sequence variants between an affected family member and control individuals from the same population, that is, our approach works by comparing a single affected individual (from a small AR family) with a number of control individuals.

Consider a set of variants within *d* basepairs of a candidate variant. In each individual, case and controls, we select homozygous variant sites from sequence *vcf* files. We distinguish only two states, *v*/*v* and “not *v*/*v*”, that is, anything other than *v*/*v*, where *v* is the variant allele (also called the alternate allele). For any two individuals, we want to measure how much their two arrays of variants differ. We do this with the Hamming distance^5^,^6^, which is the number of elements that differ between two arrays. For our set of variants and selection criteria, the two individuals can exhibit the following numbers of pairs of genotypes: *n*_1_ (*v*/*v*, *v*/*v*), *n*_2_ (*v*/*v*, not *v*/*v*), and *n*_3_ (not *v*/*v*, *v*/*v*). The Hamming distance is then equal to *n*_2_ + *n*_3_. Expressed in words, for a set of variants within *d* kb of a candidate locus, the Hamming distance between two individuals, *A* and *B*, is given by the number of homozygous variants occurring only in individual *A* plus the number of homozygous variants occurring only in individual *B*. To allow for varying numbers of basepairs flanking a candidate variant, we define a relative Hamming distance, or Hamming Distance Ratio, HDR = (*n*_2_ + *n*_3_)/(*n*_1_ + *n*_2_ + *n*_3_), which is our measure for distance between sets of variant genotypes in two individuals.

Individuals affected with a rare autosomal recessive trait tend to have parents who are related so that the two disease alleles tend to be identical by descent (IBD), that is, they are likely to be copies of one ancestral allele. Because of paucity of crossovers very close to the disease locus, SNVs in its vicinity also tend to be IBD and, thus, homozygous^3^. For this reason, we want to see whether distances between affected and control individuals are larger for true candidate variants than other candidate variants. Various approaches may be taken for such comparisons. We found the following procedure appealing and powerful. For an affected family member and *n* control individuals, we form all possible pairs of individuals and distinguish the *n* pairs containing the affected (group 1) from the *n*(*n*−1)/2 pairs consisting only of control individuals (group 2). Then we compare mean HDR values between the two groups by a one-sided *t* statistic in the expectation that, at a pathogenic variant, group 1 means exceed group 2 means. We do this for a range of values, *d* = 100 kb through 1000 kb in steps of 100 kb, and retain the largest *t* value, *t*_max_ = max_d_(*t*_d_). The limiting values of 100 and 1000 kb were chosen empirically; in our experience, the maximum *t* statistic often occurs inside this interval. In principle, the lower limit could be 0 and the upper limit could be the length of the given chromosome. The main difference would generally be increased computation time.

Candidate variants are then ranked based on their *t*_max_ value, with rank 1 corresponding to the largest *t*_max_ value. As the number of candidate variants may differ between affected individuals, we also compute the percentile (top %) of the rank of the disease variant. For the data described below, calculations were conducted by our HDR program.

While our focus is on ranking (prioritizing) candidate variants, we calculate empirical significance levels (*p*-values) associated with a given *t*_max_ statistic (candidate variant) as follows. We create null data by treating each of the *n* control individuals in turn as a pseudo-affected individual. All other individuals, including the affected, then represent pseudo-control individuals. Thus, we construct *n* null datasets, each consisting of 1 pseudo-affected and *n* pseudo-controls in analogy to 1 affected and *n* controls in the observed data. In each of the *n* null datasets, we perform the analysis done on the observed data. For a given candidate variant, the *p*-value associated with the observed *t*_max_ statistic is computed as the proportion of null datasets with a null-*t*_max_ value at least as large as the observed *t*_max_ value. To be conservative, we include the observed data with the null-data, so the smallest possible significance level is *p* = 1/(*n* + 1). These statistical analyses can be performed with a suitably modified version of the *maxstat* program written in Pascal.

## Results

**Figure 1:**
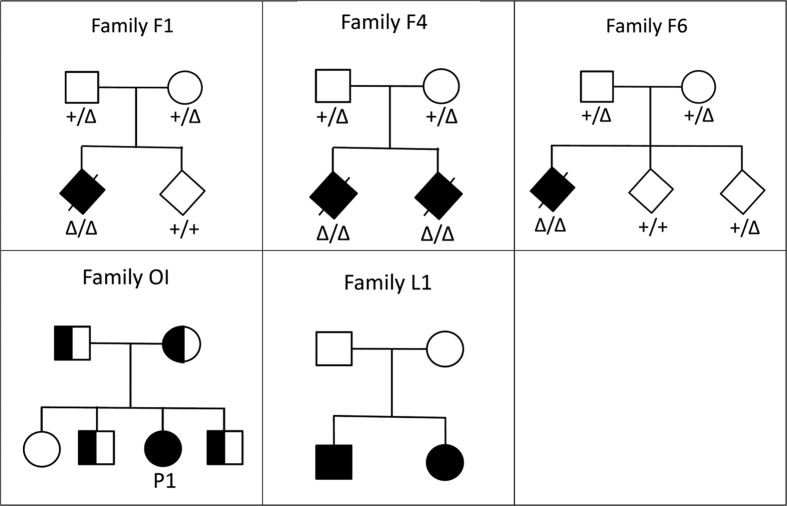
Pedigree drawings for families F1, F4, F6, OI, and L1. For families F1, F4, and F6, genotypes are marked for individuals with DNA available and tested; the following abbreviations are used: +, normal (wild-type) variant; Δ, rare mutant variant. Family OI: The affected individual (P1) is indicated with a solid symbol, heterozygotes are shown with half-solid symbols.

As a proof of concept, we applied our approach to six patients from five different small AR families (Fig. 1) for which the disease-causing mutation had previously been identified and published^7^,^8^,^9^,^10^,^11^,^12^,^13^,^14^ (Table 1). Families F1, F4, F6, and OI are members of the French-Canadian population of Québec, which originates from approximately 8500 French settlers who immigrated more than 300 years ago^15^. Family L1 is of European ancestry. For our approach to be valid it is important that control individuals be from the same ethnic background as the patients. For families F1, F4, and F6, we chose 30 members of this same population as controls; for family OI, we had 32 control individuals available; and for family L1 we used 30 European control individuals. As our approach currently works with one affected individual versus a number of controls, the two affected individuals in family L1 were considered separately. All control individuals had previously been investigated for reasons unrelated to the patients. All individuals, cases and controls, had been exome-sequenced at McGill University and Genome Québec Innovation Center, Montreal, after obtaining approval from the Institutional Review Board of McGill University and informed consent from all individuals. The detailed sequencing protocol is given in the Methods section. To narrow down the list of variants, we applied several filtering steps as follows: We selected exonic (missense, nonsense, and Indels) or splicing or UTR variants annotated as homozygous or possibly homozygous with an allele frequency <5% in 1000 Genome and EVS databases, then removed variants with quality scores less than 50 or map quality score less than 20, read depths less than 5, and those that were seen in more than 10 individuals in our exome database of ~1200 samples. These filtering steps resulted in the *m* = 10 through 50 final candidate variants shown in Table 1.

Results obtained by our family-control analysis demonstrate that we were able to prioritize the known disease variants to be in the top 2% to 10% of candidate variants (Table 1), that is, the HDR method narrowed down the original number of shortlisted candidate variants to between 10-fold and 50-fold smaller lists.

The *p*-values for the test statistic of the true disease variant ranged from 0.0303 through 0.0645. Combining five independent *p*-values (using only one individual in family L1) by the Fisher method^16^,^17^ (*pvalues* program) results in a final empirical significance level of 0.0013. Thus, we demonstrated significantly larger distances between case and control individuals for homozygous pathogenic variants than non-pathogenic variants.

## Discussion

Our approach has several advantages over existing (homozygosity mapping, HM) methods: (1) Our HDR method can provide a ranking of homozygous regions while HM approaches rank on the basis of ROH length, which is less reliable. (2) We can assess inherited regions specific to disease pedigrees more accurately than using heterogeneous populations by using relatively homogenous control individuals in the same population as family members. (3) Most HM approaches work with sliding windows of a given size and additional parameters like minimum number of SNPs in an ROH, minimum length of an ROH, and maximum number of heterozygous SNPs in an ROH. These settings may or may not be optimal; on the other hand, our HDR method employs a single estimated parameter for prioritizing candidate variants. Thus, the HDR approach does not require any parameters that need to be fixed at the outset. A limitation of our approach is that control individuals are required while HM may be carried out on single (affected) individuals. We used 30 control individuals as a compromise between cost and efficiency, (1) because our approach proved successful with the given numbers, and (2) to obtain a p-value potentially smaller than 0.05 given that we include the observed data in our null data. Applied to our six patients and corresponding control individuals, the HDR method narrowed down the original number of shortlisted candidate variants to more than tenfold smaller lists.

## Methods

### Pathogenic mutations

#### Families F1/F4/F6

A pathogenic variant for multiple intestinal atresia^18^ was found in three affected individuals from three different families. It is a homozygous mutation for a four-base intronic deletion on chromosome 2 at positions 47,221,651-47,221,654 in the TTC7A gene, immediately adjacent to a consensus GT splice donor site.

#### Family OI

A pathogenic variant for osteogenesis imperfecta^12^ was observed in an affected individual. It is a homozygous missense mutation, T > C, at position 22,058,957 on chromosome 8 in the UTR3 region of the BMP1 gene.

#### Family L1

A pathogenic variant for leukodystrophy^13^ was observed in an affected brother-sister pair in this small family. It is a missense mutation, rs138249161, at position 106,432,421 on chromosome 12 in the POLR3B gene.

### Sequencing protocol

Whole exome library preparation, capturing, sequencing and bioinformatics analyses were performed at the Genome Québec Innovation Center, Montreal, Canada, as detailed in our previous publications (see main text). In brief, 3 micrograms of DNA of 65 individuals were used for exome capture and sequencing. For each exome, the Burroughs Wheeler alignment (BWA) version 0.5.9^19^ was used to align the sequencing reads (100 bp paired-end) to the human reference sequence (hg19). Alignments were converted with SamTools^19^ from SAM format to sorted, indexed BAM files. Regions surrounding potential indels were realigned with the GATK IndelRealigner tool^20^. Picard-tools were used to remove invalid alignments and duplicate reads from the BAM files^19^. Single nucleotide variant (SNV) and indel variants were called with Samtools (v. 0.1.17) mpileup and were then quality filtered so that at least 20% (SNVs) or 15% (Indels) of reads supported the variant calls. All called variants were annotated with the ANNOVAR program^21^ to identify exonic or splicing or UTR variants, allele frequency in the 1000 Genomes Project^22^, Exome Variant Server (EVS, version 6500) and dbSNP (version 135), SIFT, PolyPhen-2 and PHASTCONS scores.

## Web Resources

HDR program

http://www.gi.med.osaka-u.ac.jp/software/hdr/

ANNOVAR Software

http://www.openbioinformatics.org/annovar/

Exome Variant Server, NHLBI GO Exome Sequencing Project (ESP) http://evs.gs.washington.edu/EVS/

GATK Software

http://www.broadinstitute.org/gsa/wiki/index.php/The_Genome_Analysis_Toolkit

Maxstat program

http://lab.rockefeller.edu/ott/programs

PVALUES program

http://www.jurgott.org/linkage/util.htm#pvalues

## Additional Information

**How to cite this article**: Imai, A. *et al.* Beyond Homozygosity Mapping: Family-Control analysis based on Hamming distance for prioritizing variants in exome sequencing. *Sci. Rep.* **5**, 12028; doi: 10.1038/srep12028 (2015).

## Supplementary Material

Supplementary Information

## Figures and Tables

**Table 1:** Results of our family-control analysis for prioritizing *m* final candidate variants in an affected individual from five families.

**Family**	**Gene**	**Disease**	**rank**	***m***	**%**	***p***
F1	TTC7A	Multiple intestinal atresia	1	10	10.0	0.0645
F4	TTC7A	Multiple intestinal atresia	1	14	7.1	0.0645
F6	TTC7A	Multiple intestinal atresia	1	18	5.6	0.0645
OI	BMP1	Osteogenesis imperfecta	1	14	7.1	0.0303
L1a	POLR3B	Leukodystrophy	1	50	2.0	0.0645
L1b	POLR3B	Leukodystrophy	4	44	9.1	0.0645

Rank = order of test statistic (largest *t*_max_ ranked 1) for pathogenic variant among the *m* candidate variants; % = top percentile rank, 100 × rank/*m*; *p* = empirical significance level. L1a and L1b refer to two affected individuals in family L1.
