# Supplementary material for: Beyond Homozygosity Mapping: Family-Control analysis based on Hamming distance for prioritizing variants in exome sequencing
Source: Sci Rep. 2015 Jul 6;5:12028. doi: 10.1038/srep12028 (PMC5155624; doi:10.1038/srep12028)
Supplement: Supplementary Information [file srep12028-s1.pdf]

Beyond Homozygosity Mapping: Family-Control analysis based on Hamming distance for prioritizing variants in exome sequencing

Atsuko Imai, Akihiro Nakaya, Somayyeh Fahiminiya, Martine Tétreault, Jacek Majewski, Yasushi Sakata, Seiji Takashima, Mark Lathrop, Jurg Ott

Supplementary Information

For each of the six individuals with known pathogenic variants (see Table 1 of main text), Table S1 below shows all shortlisted candidate variants used in our HDR calculations. The headings have the following meaning:

Group 1, group 2: Mean HDR values for the two types of pairs of individuals (group 1 = affected-control, group 2 = control-control)

n1, n2: Numbers of pairs in groups 1 and 2, respectively

Teststat: Statistic (t_{max}) for ranking candidate variants

R: Distance (100 kb through 1000 kb in steps of 100 kb) from candidate variant at which maximum *t* statistic was obtained

p: Empirical significance level associated with t_{max} statistic

chr, position: Chromosomal position of candidate variant

Table S1

List of all shortlisted candidate variants in each of the six patients used in our HDR calculations

Family F1

Group 1	Group 2	n1	n2	Teststat	R	p	chr	position
0.9461	0.7446	30	435	21.4280	400	0.0645	2	47221651
0.9325	0.7117	30	435	13.4096	400	0.0645	2	96780987
0.6404	0.5448	30	435	4.0005	300	0.3871	3	63898266
0.6317	0.4648	30	435	7.0371	100	0.0323	4	145041707
0.6317	0.4648	30	435	7.0371	100	0.0323	4	145041707
0.8197	0.8206	30	435	-0.0454	700	0.9032	7	1477775
0.6766	0.7302	30	435	-1.0182	400	0.6774	7	19184751
0.7066	0.7197	30	435	-0.5223	600	0.6774	7	100639356
0.8207	0.8073	30	435	0.4140	400	0.6129	10	17891606
0.8940	0.6773	30	435	6.1923	100	0.2581	14	24646407

Family F4

Group 1	Group 2	n1	n2	Teststat	R	p	chr	position
0.6287	0.6591	30	435	-2.2488	900	0.9677	1	3753032
0.9582	0.7446	30	435	23.8125	400	0.0645	2	47221651
0.7182	0.6528	30	435	1.7252	300	0.5484	2	113145900
0.9306	0.5809	30	435	10.6393	300	0.1290	4	110384050
0.7493	0.7276	30	435	1.2184	400	0.6774	5	64070616
0.8837	0.7848	30	435	5.1549	600	0.3226	9	123787697
0.8357	0.8397	30	435	-0.1571	600	0.8065	10	18439797
0.5274	0.5270	30	435	0.0155	100	0.7419	11	64112394
0.5921	0.6157	30	435	-0.7640	100	0.8065	13	42733389
0.8817	0.6773	30	435	7.6355	100	0.1613	14	24646408
0.8862	0.7873	30	435	4.6546	100	0.2581	18	7023207
0.6718	0.7360	30	435	-2.0901	300	0.8710	20	271204
0.8620	0.6569	30	435	6.6706	400	0.3871	21	34003927
0.7967	0.7301	30	435	2.4446	200	0.3226	23	47307172

Family F6

Group 1	Group 2	n1	n2	Teststat	R	p	chr	position
0.8324	0.7275	30	435	5.7272	100	0.2581	1	242042364
0.8102	0.6619	30	435	12.9711	900	0.0645	1	248637367
0.8102	0.6619	30	435	12.9711	900	0.0645	1	248637367
0.9499	0.7446	30	435	22.5812	400	0.0645	2	47221651
0.9667	0.7400	30	435	6.1658	100	0.1935	4	140811063
0.5757	0.5493	30	435	1.2226	500	0.4839	8	145487205
0.6831	0.5809	30	435	3.3563	100	0.2903	9	140007244
0.6549	0.5913	30	435	1.2926	100	0.5161	13	77566213
0.6589	0.6496	30	435	0.4356	500	0.6452	15	41228471
0.8985	0.8291	30	435	2.4174	100	0.4516	17	44408458
0.7519	0.7993	30	435	-1.8598	1000	0.8710	20	46279830
0.9418	0.8375	30	435	3.6471	300	0.4194	23	22291631
0.7779	0.7176	30	435	2.0803	300	0.4516	23	70472939
0.6687	0.4763	30	435	7.1048	600	0.1613	23	105190439
1.0000	0.7990	30	435	14.6270	700	0.0323	23	132092479
0.6344	0.6982	30	435	-1.1260	300	0.6129	23	136648899
0.6447	0.6084	30	435	1.4293	400	0.4839	23	153033339
0.7106	0.5726	30	435	9.0898	900	0.2581	23	153581714

Family L1, individual *a*

Group 1	Group 2	n1	n2	Teststat	R	p	chr	position
0.5844	0.6049	30	435	-0.6582	100	0.8387	1	156844800
0.7808	0.7432	30	435	2.0716	700	0.3871	1	157504466
0.7417	0.6729	30	435	5.8050	500	0.0968	1	160187324
0.4584	0.5251	30	435	-1.1984	100	0.6129	1	197026151
0.7355	0.6651	30	435	3.7270	100	0.3548	1	201186501
0.8756	0.7352	30	435	7.0622	100	0.1290	1	205211021
0.7084	0.6500	30	435	1.5057	200	0.5161	2	26403750
0.8155	0.7103	30	435	3.0008	100	0.3548	2	44545234
0.8155	0.7103	30	435	3.0008	100	0.3548	2	44545234
0.5679	0.6141	30	435	-1.1661	400	0.5484	2	86304932
0.7985	0.7307	30	435	1.6027	100	0.5484	2	191231418
0.7963	0.7872	30	435	0.6956	400	0.6774	3	186575720
0.8812	0.7902	30	435	6.9479	200	0.1290	3	195295708
0.7877	0.7386	30	435	2.8254	400	0.3871	3	195514904

0.7697	0.7226	30	435	3.3279	500	0.5484	5	79435419
0.5098	0.4927	30	435	1.5193	600	0.5484	5	176981495
0.8594	0.8352	30	435	1.2375	700	0.5484	6	1390137
0.8969	0.8481	30	435	4.5034	800	0.3548	6	4079250
0.7506	0.7560	30	435	-0.1811	100	0.5806	6	148865088
0.7895	0.5742	30	435	14.5781	400	0.0323	6	157099185
0.6098	0.5041	30	435	5.5257	100	0.1290	8	24211992
0.7435	0.8459	30	435	-1.9055	300	0.8710	8	105601198
0.8149	0.7680	30	435	4.3772	1000	0.4194	9	36216324
0.7606	0.7172	30	435	4.6728	1000	0.3548	9	138899381
0.7191	0.7896	30	435	-2.2728	300	0.9677	9	141107551
0.5885	0.5799	30	435	0.2888	300	0.3871	11	100863593
0.6671	0.6075	30	435	2.7809	200	0.5806	12	3919812
1.0000	0.7481	30	435	14.8125	200	0.0645	12	106826199
0.9088	0.8071	30	435	2.5292	100	0.4516	12	108168740
0.9054	0.8272	30	435	5.7386	500	0.2903	12	110290224
0.9000	0.7310	30	435	10.3874	700	0.0968	12	110969768
0.6298	0.6157	30	435	0.2967	100	0.5484	13	20247226
0.7019	0.7509	30	435	-1.2408	200	0.9032	13	50275349
0.7167	0.7091	30	435	0.2786	200	0.5484	14	77737364
0.8442	0.7578	30	435	6.8167	800	0.2258	14	91699763
0.9022	0.8827	30	435	1.8237	800	0.5484	14	95566108
0.7417	0.6729	30	435	4.6065	600	0.2903	14	104647113
0.6904	0.6489	30	435	3.5799	800	0.4194	17	7750177
0.4094	0.2732	30	435	5.7728	100	0.4194	17	38296601
0.7911	0.7360	30	435	4.6277	1000	0.3871	19	1941176
0.9349	0.6459	30	435	8.9682	100	0.1935	19	12810274
0.9349	0.6459	30	435	8.9682	100	0.1935	19	12810274
0.6614	0.6394	30	435	0.6876	100	0.6452	20	43343899
1.0000	0.8190	30	435	11.7647	100	0.0645	22	39131509
0.8265	0.7878	30	435	1.0687	400	0.3226	23	11160419
1.0000	0.8448	30	435	10.4183	100	0.0968	23	38156584
0.7174	0.6901	30	435	0.7256	500	0.7419	23	70389354
0.7375	0.7539	30	435	-0.4694	800	0.5806	23	127186133
0.6376	0.6034	30	435	2.3868	500	0.5161	23	151092264
0.8511	0.6460	30	435	11.2674	200	0.2258	23	153762615

Family L1, individual *b*

Group 1	Group 2	n1	n2	Teststat	R	p	chr	position
0.7465	0.7080	30	435	3.1738	900	0.3548	1	20971158
0.6045	0.4531	30	435	8.9011	100	0.1613	1	22165560
0.7811	0.7341	30	435	2.0665	500	0.4194	1	28261738
0.6588	0.4796	30	435	6.0033	100	0.1935	1	29063346
0.9394	0.4611	30	435	14.3510	100	0.0323	1	43849952
0.7932	0.6118	30	435	8.7779	100	0.1613	1	44360114
0.7391	0.7453	30	435	-0.2797	800	0.5806	1	157504466
0.7686	0.7245	30	435	1.2553	100	0.5806	1	242052841
0.5909	0.5581	30	435	1.9022	1000	0.5161	1	248201550
0.8042	0.7248	30	435	2.0547	200	0.4194	2	44545234
0.7871	0.7289	30	435	4.1082	1000	0.2258	2	191231418
0.9026	0.8690	30	435	1.4587	200	0.5161	2	242707650
0.8062	0.7433	30	435	1.0250	100	0.3548	5	140230792
0.7273	0.7135	30	435	0.7056	1000	0.7097	7	100271438
0.9841	0.6408	30	435	14.0453	300	0.1935	8	53045842
0.6862	0.6628	30	435	0.4285	200	0.6129	8	86126829
0.8380	0.8000	30	435	3.6996	800	0.2581	9	139235823
0.9550	0.8900	30	435	3.3489	100	0.7419	10	80828849

0.7472	0.6154	30	435	7.7405	700	0.1935	10	93389538
0.7516	0.7317	30	435	1.3233	800	0.5161	10	95462279
1.0000	0.7481	30	435	14.8125	200	0.0645	12	106826199
0.9258	0.7310	30	435	13.3464	700	0.0968	12	110969768
0.6093	0.6034	30	435	0.1272	200	0.5484	13	20247226
0.5888	0.5521	30	435	3.3216	900	0.3226	14	23345962
0.6882	0.3392	30	435	16.6739	100	0.1613	16	27801147
0.5451	0.4976	30	435	2.7014	400	0.3871	16	31004013
0.7658	0.6849	30	435	3.6819	700	0.5484	17	55197982
0.8468	0.8082	30	435	2.6532	600	0.5161	17	76109096
1.0000	0.6745	30	435	17.2313	100	0.0323	18	13029951
0.6854	0.6615	30	435	1.8106	1000	0.5806	18	21723319
0.7618	0.6917	30	435	3.2976	200	0.2903	18	22056759
0.5727	0.5304	30	435	1.2906	300	0.7419	18	49866783
0.7397	0.7650	30	435	-0.8193	200	0.7419	19	17397501
0.8229	0.7969	30	435	0.6619	100	0.7419	20	10579398
0.7653	0.7673	30	435	-0.0605	200	0.7742	20	33814576
0.8340	0.8721	30	435	-1.2517	400	0.9355	20	34817990
0.9211	0.8097	30	435	7.1906	500	0.1935	20	43535225
0.8557	0.7473	30	435	7.9896	200	0.2581	20	48121370
0.8793	0.6826	30	435	11.7602	100	0.1290	21	45648099
0.7843	0.7830	30	435	0.0487	100	0.6129	21	46924440
0.7235	0.5810	30	435	4.4987	200	0.1935	22	41076970
0.9075	0.7056	30	435	16.4994	800	0.2581	22	41971076
0.9141	0.8520	30	435	5.6386	1000	0.3548	22	42950004
0.8398	0.7951	30	435	2.4476	100	0.4516	22	43525570

Family OI

Group 1	Group 2	n1	n2	Teststat	R	p	chr	position
0.5661	0.5863	32	496	-0.4298	100	0.9091	1	25648421
0.9175	0.8595	32	496	3.5327	400	0.4848	5	796277
0.7918	0.7481	32	496	1.4799	1000	0.4848	5	140309125
0.7816	0.6131	32	496	4.6320	100	0.1818	6	18222560
0.8187	0.7894	32	496	1.4628	900	0.5152	7	37960285
0.8972	0.6440	32	496	23.1354	100	0.0303	8	22058957
0.8665	0.7697	32	496	9.2965	800	0.1818	8	22424180
0.6844	0.6048	32	496	6.2798	1000	0.2727	10	121410904
0.7380	0.6719	32	496	4.1562	600	0.3939	11	44286142
0.5837	0.5597	32	496	1.7622	1000	0.7273	12	7342367
0.5837	0.5597	32	496	1.7622	1000	0.7273	12	7342367
0.7176	0.6728	32	496	2.6734	800	0.6667	12	8693431
0.5631	0.6480	32	496	-2.0950	100	0.9091	15	89400023
0.9008	0.6055	32	496	21.2759	500	0.0303	16	23478708
